# Efficient Collection of Skin Biopsies Using the Tissue Sampling Unit^®^ for Subsequent Cryopreservation and Culture of Fibroblasts

**DOI:** 10.3390/mps8050114

**Published:** 2025-10-01

**Authors:** Phillip H. Purdy, Bethany Redel, Paula Chen, Ashley J. Rahe, Aashi Jivan, Scott F. Spiller

**Affiliations:** 1 USDA ARS National Animal Germplasm Program, 1111 S. Mason St., Fort Collins, CO 80521, USA; ashley.rahe@usda.gov (A.J.R.); aashi.jivan@usda.gov (A.J.); scott.spiller@usda.gov (S.F.S.); 2 USDA ARS Plant Genetics Research Unit, 920 E. Campus Dr., University of Missouri, Columbia, MO 65211, USA; bethany.redel@usda.gov (B.R.); paula.chen@usda.gov (P.C.)

**Keywords:** Tissue Sampling Unit, cryopreservation, fibroblast, somatic cell nuclear transfer, genotype, genebank, germplasm

## Abstract

Dermal tissue samples are a rich source of germplasm because they can be readily collected, frozen as part of a genebank collection, digested and cultured, and used for a variety of purposes such as genotyping or other forms of genetic research. Derived fibroblasts can also be used for somatic cell nuclear transfer, and the remaining cells can be frozen for future use. However, collection of tissues with ear notchers, scalpels, or biopsy punches can be problematic because tissue handling and the tool surfaces can contaminate the samples. Therefore, the modification of the Allflex Tissue Sampling Unit (TSU) system was explored to determine if the technology can empower rapid collection of clean samples that are easily identifiable and portable. Results indicate that the TSU system was efficient, and samples that were collected and processed for tissue culture resulted in successful derivations of fibroblasts from 7 of 11 animals. Thus, the TSU system appears to be a viable option for collecting and preserving dermal tissue for genebanking and other applications where simple, rapid collection of large quantities of samples is required.

## 1. Introduction

The USDA ARS National Animal Germplasm Program (NAGP) is a genebank containing samples of germplasm (e.g., semen, eggs, embryos, DNA, tissue, blood, organs, etc.) from agricultural species. The purpose of the NAGP is to collect and maintain a collection of genetic resources so that it can be used for research (e.g., genotyping, genetic improvement) or repopulation (e.g., following genetic bottlenecks or depopulation events) when needed. To that end, one of the most dynamic forms of germplasm is a tissue biopsy from any mammalian species because it can be easily collected, frozen, and the fibroblasts derived following digestion can be used for somatic cell nuclear transfer (SCNT) or reprogrammed to create induced pluripotent stem cells. Moreover, once fibroblasts have been derived and a subsample used for SCNT, the remaining fibroblasts can be frozen for future culture and use. This results in an abundant supply of genetic resources from a single collection which can be frozen, thawed, and cultured, and derived cells can be frozen again multiple times.

Traditionally, tissue samples have been collected in the form of ear notches, but the device to collect the samples can be cumbersome; the tissue can be contaminated or lost, and often the sample size and quality using this method are inadequate. Groeneveld et al. [1] recognized these issues and were able to overcome the challenges using an integrated tagging and vial system for collecting pigs, sheep, and goats, but that system is no longer available. Recently, Allflex (Tissue Sampling Unit—Allflex) created a Tissue Sampling Unit (TSU) for the collection of samples for genetic testing purposes, while simultaneously applying ear tags if desired, in which the ear punch sample is placed directly into a tube containing a proprietary preservative solution. The benefits of this method and equipment are that it is easy to operate, and a clean sample is collected which can be kept at room temperature for up to a year, or frozen for longer term storage and thawed when needed. However, the TSU preservative does not allow samples to be collected for use in SCNT applications. Consequently, the purpose of our research was to determine if the TSU could be successfully adapted to collect ear punches intended for SCNT or uses where the cells and tissue must remain viable.

## 2. Experimental Design

Samples from adult sheep (n = 9) and lambs (<1 yr of age, n = 2) were collected on farm, placed in insulated shipping containers to maintain the temperature at 5 °C, and transported via commercial carrier to the laboratory within 24 h of collection. Upon arrival, the samples were frozen and stored in liquid nitrogen, and when available, replicate samples were held at 5 °C for up to 3 d. After 1 month of storage, the samples were thawed, digested, and the resulting cells were placed in tissue culture. Samples were then evaluated for cell quantity upon reaching confluence.

### 2.1. Materials: Reagents and Reagents Set Up

Holding Medium composed of M199 Medium containing 25 mM Hepes buffer, 2 mM L-glutamine, and 0.15% (*w*/*v*) sodium bicarbonate (M199; Gibco 11150-059), and supplemented with 100 units/mL penicillin G (ThermoFisher Scientific, Waltham, MA, USA, J63032.22), and 100 µg/mL gentamicin sulfate (Sigma Aldrich, St. Louis, MO, USA, product # G1272) [2]. Freeze for long term storage or at 5 °C for short term storage (less than 2 d).

Tissue Cryopreservation Medium [2] is composed of Holding Medium supplemented with 2 M glycerol (14.6% by volume using sterile glycerol).

TES-Calcium (TESCA) buffer for dilution of collagenase (50 mM TES (ThermoFisher Scientific B21819.30), 0.36 mM Calcium chloride, pH 7.4). A 100 mg bottle of collagenase (200 U/mL type IV, Sigma C5138) contains 12,500 Units and is diluted in 100 µL TESCA buffer [3].

Sodium chloride (NaCl) solution for dilution of DNAase I (25 Kunitz/mL, Sigma D-4263). The NaCl is prepared at 0.15 M and the DNAase I is soluble at 5.0 mg/mL Therefore, 1 vial (0.25 mg bottle) diluted with 1 mL of buffer contains 2000 Kunitz/mL.

Digestion Medium for 50 mL

M199 (high glucose, no L-glutamine, ThermoFisher #11960-044)40.695 mL15% FBS (Fetal Bovine Serum, ThermoFisher #10437-028) 7.5 mLCollagenase (10,000 units)80 µLDNAase I (1250 Kunitz)0.625 mLAntibiotics (1 μg/mL gentamicin, 10mg/mL stock, Sigma G1272)100 µLAntibiotic-Antimycotic (100×, ThermoFisher #15240062)1 mL

Culture Medium, for 50 mL

M19933 mL15% FBS 15 mL1% (1X) GlutaMAX (ThermoFisher #35050-061)1 mLAntibiotic-Antimycotic 1 mL

Additional media

10% betadine solution

70% ethanol

Hanks Balanced Salt Solution (HBSS, ThermoFisher Product number 14170)

Trypsin EDTA (Sigma Aldrich T4049)

### 2.2. Equipment

Tissue Sampling Unit preparation

Tissue Sampling Units (Allflex, No Liquid, Product Number 21315472)

3 mm stainless steel ball bearings

Tissue Sampling Unit Decapper (Allflex, Product Number 21315403)

Tissue Sample Unit Applicator (Allflex)

Tissue digestion and culture

Disposable Petri dish

Scalpel

1 mL pipette and tips

15 mL tubes

24-well plate or 5 mL vented flask

Fine forceps

## 3. Procedure—Collection, Shipping, Cryopreservation, Thawing, Digestion, Culture, Counting

### 3.1. Tissue Sampling Unit (TSU) Preparation

**Figure 1:**
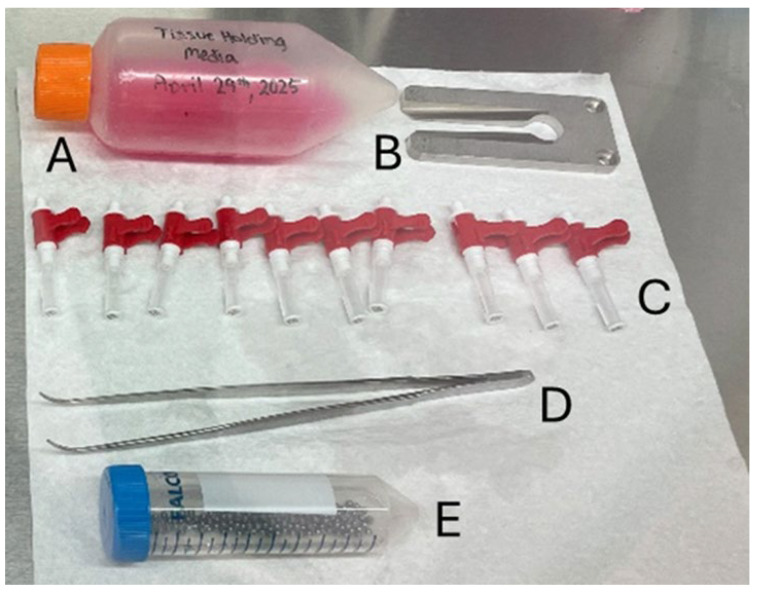
Equipment for preparation of tissue sampling units (TSU). The equipment includes the Holding Medium (A), the decapper (B), empty TSUs (C), forceps (D), and 3 mm ball bearings in 70% ethanol (E).

**Figure 2:**
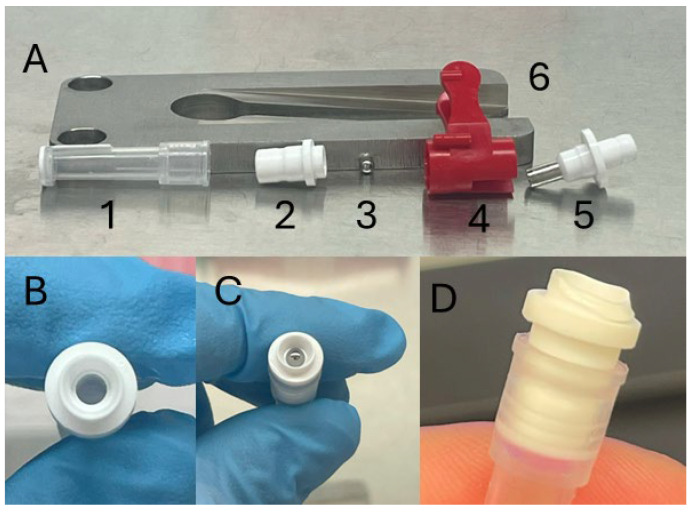
Tissue sampling unit (TSU) components. Panel (**A**) depicts the collector (1), connector (2) ball bearing (3), blade apparatus (4 and 5), and decapper (6). Panels (**B**–**D**) illustrate empty connectors, a connector sealed with a ball bearing, and a damaged connector which should not be used, respectively.

**Figure 3:**
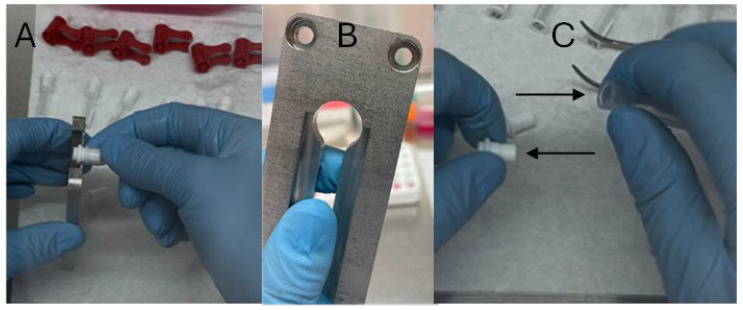
Separation of the tissue sampling unit into its constitutive parts. The image depicts removal of the connector (**A**) using the decapper (**B**) into the two separate components (arrows identified in (**C**)).

**Figure 4:**
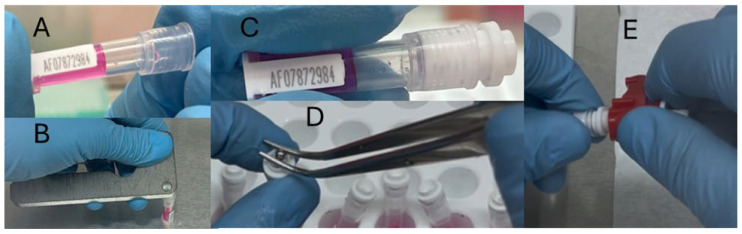
Recapping of tissue sampling units (TSU). Panel (**A**) depicts a TSU filled with Holding Medium which is then sealed using the decapping tool (**B**). A sealed TSU is depicted in panel (**C**) which is then sealed with a ball bearing (**D**) and finally completely reassembled (**E**).

- CRITICAL STEP: All steps within this area of preparation should be conducted using sterile technique in a biosafety cabinet. Refer to Figure 1 for examples of the parts.
- Place 3 mm ball bearings in 70% ethanol.
- CRITICAL STEP: Ball bearings are not needed if the standard type TSUs are used that were originally intended for genotyping and contained the proprietary medium from Allflex are used. In this instance the sealing ball is included with the TSU, and it is critical to empty the proprietary medium, wash and rinse the TSU with distilled and deionized water, and finally sanitize the TSU components with 70% ethanol.
- Remove TSU blade (Figure 2A(4 and 5)) from TSU and place in 70% ethanol.
- Separate tissue collectors (Figure 2A(1)) from TSU connectors (Figure 2A(2)) and place both in 70% ethanol.
- After 10 min, remove all parts from ethanol and allow them to completely dry.
- Add 200 µL of Holding Medium to each of the tissue collectors (Figure 3A).
- Seal the tissue collector with the connector (Figure 4B–D).
- Insert a single ball bearing into the opening of the TSU connector and ensure it is firmly secured using the blade holder (Figure 2A(4)). Repeat for all connectors prior to subsequent steps.
- Attach the blade (Figure 2A(4 and 5)) to the TSU (Figure 4E).

### 3.2. Animal Preparation and Collection

- Trim the hair or wool with fine electric clippers and ideally shave the location for the biopsy from both sides of the ear where the sample will be collected.
- Wash both sides of the ear with 10% betadine and then 70% ethanol. Allow the ear to dry.
- Collect the samples per the TSU instructions (Tissue Sampling Unit—Allflex).
- Store the samples at 5 °C.

### 3.3. Overnight Transportation

- Expedited, overnight shipping from the collection location to the processing laboratory is ideal. However, samples can be held for up to 3 d at 5 °C following collection with the TSU method.
  ○ CRITICAL STEP: Samples have been held for longer than 3 d, but the quality declines rapidly the longer the samples are stored prior to cryopreservation or tissue culture.
- For transportation, place the samples in a shipping cooler with multiple reusable ice packs ensuring the internal temperature will remain at 5 °C during transportation.
  ○ CRITICAL STEP: Pack the shipping cooler so that the samples do not come in direct contact with the reusable ice packs. Samples should be separated using multiple layers of insulated material or bubble wrap.

### 3.4. Cryopreservation

- Place cryovials labeled with animal identification numbers into a rack in a Styrofoam box containing liquid nitrogen.
  ○ CRITICAL STEP: Each tube must contain at least 1.5 mL of liquid nitrogen.
- Samples diluted in Holding Medium and cooled should be warmed to room temperature (22 to 23 °C).
- Transfer the individual tissue samples to 0.5 mL Cryopreservation Medium at 22 °C and ensure the tissue is fully submerged.
  ○ CRITICAL STEP: To increase workflow and productivity, Cryopreservation Medium can be placed in the wells of a 48-well plate. Each well can then be used for a single sample.
  ○ CRITICAL STEP: Only transfer the tissue and not the Holding Medium.
- Incubate the samples for 5 min at 22 °C.
- Remove the tissue from the Cryopreservation Medium and blot dry.
- Drop the samples into the liquid nitrogen in the respective, labeled cryovials.
  ○ CRITICAL STEP: Only 1 sample is vitrified in a cryovial to avoid samples sticking together during the process. However, multiple samples can be stored in the same cryovial after vitrification, if desired.
- Liquid nitrogen should be poured off from the sample and the tube capped to avoid liquid nitrogen expansion and maintain sample integrity.
  ○ CRITICAL STEP: Samples should not be out of liquid nitrogen vapor for longer than 6 s.
- Store samples in liquid nitrogen or in a liquid nitrogen vapor tank.

### 3.5. Thawing

- Remove the cryovials containing the samples from liquid nitrogen storage and place the cryovial in a 22 °C water bath for 10 min.
  ○ CRITICAL STEP: ensure only the tube, and not the actual samples contact water.
  ○ CRITICAL STEP: agitate samples in water bath to create a uniform thaw.
  ○ CRITICAL STEP: sanitize the tube with 70% ethanol before placing it in the biosafety cabinet.
- Remove the sample from the cryovial and incubate the tissue in 5 mL of M199 for 5 min at 22 °C.

### 3.6. Tissue Culture

- The tissue culture protocol was adapted from Progeria Research Foundation https://www.progeriaresearch.org/fibroblast-cell-culture-protocols/, accessed on 10 September 2025)
- Tissue culture must be performed in a biosafety cabinet using sterile technique.
- Place the sample in a disposable Petri dish and mince the tissue with a scalpel until the tissue resembles a pulp.
- Dilute sample with 5 mL of Digestion Medium and transfer to a 15 mL tube.
- Rinse the Petri dish with 5 mL of fresh Digestion Medium and transfer to the 15 mL tube containing the sample.
- Incubate at 37 °C for 4–6 h.
- Agitate the sample often using a vortex mixer at room temperature and return to the incubator as quickly as possible.
- Centrifuge the samples at 1000× *g*.
- Remove the supernatant.
- Wash the sample 3× with 3 mL of Culture Medium for each wash.
- Remove the final supernatant.
- Dilute the final pellet with Culture Medium.
  ○ CRITICAL STEP: the final pellet can be diluted with either 0.7 or 5 mL of Culture Medium. The smaller volume can be cultured in a 24-well plate whereas the larger volume can be cultured in a 5 mL vented flask.
- Culture the samples at 37 °C in 5% CO_2_ in humidified air for 10 d or until the fibroblasts reach 80% confluence.
- Culture Medium (one-third of the total volume of the well or flask) should be refreshed every 24–48 h.
- At 80% confluence, remove the Culture Medium and wash the fibroblasts with HBSS in the well/flask.
- Add Trypsin EDTA to the well/flask (0.3 mL/1 mL) and incubate (37 °C in 5% CO_2_) for 3–5 min.
- Tilt the plate/flask to loosen the fibroblasts. Gentle pipetting is also acceptable.
- Transfer the sample to a centrifuge tube (2 mL or 10 mL depending on culture volume) and dilute with Culture Medium.
- Rinse the plate/flask with additional Culture Medium and combine with the sample so that the final volume is 1.8 or 10 mL (plate or flask).
- Wash the samples 3 X at 1000× *g* using fresh Culture Medium each time.
- Suspend the pellet in a small volume of Culture Medium.
- Count the fibroblasts using a hemocytometer or flow cytometer to determine the final quantity.
- Successful derivations can now be used for SCNT or frozen for later use.

## 4. Expected Results

Collection, preservation, and utilization of animal dermal tissues hold great promise for genebanking and repopulation activities. Ear notches are a high-quality source of germplasm because they can be used for genetic analyses or, following extraction and digestion, the resulting fibroblasts can be used for SCNT or other molecular biology applications. The protocols for collection, preservation, and tissue culture are well established and should routinely result in successful derivation of fibroblasts.

**Figure 5:**
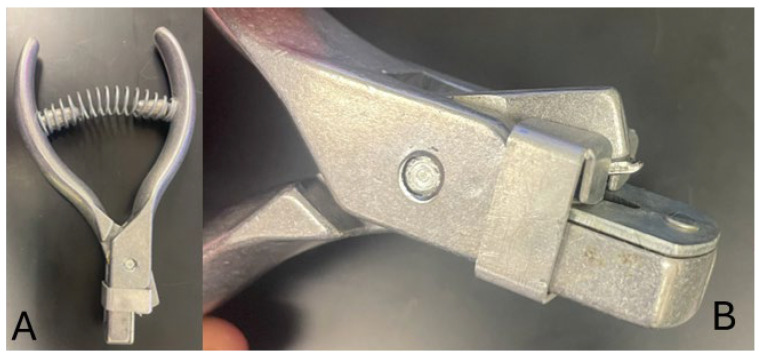
Images of ear notcher; complete device (**A**) and close-up of cutting edge (**B**).

The most problematic area of these processes is the collection methodology. While ear notchers (Figure 5) are routinely used for marking animals, they are challenging to use. Often the tissue is lost upon collection, the notcher requires cleaning between animals, and some producers do not want unsightly marks along the edges of their animals’ ears. Biopsy punches can be a viable alternative, but that form of blade is not safe for the animal or the person collecting the tissue. Furthermore, from the genebank development perspective, neither the notcher nor the biopsy punch minimizes the chance of contamination of the sample. In both instances the tissue is exposed to a variety of surfaces and environments when transferring to a storage tube, which increases the probability of sample contamination from bacteria or mycoplasma thus diminishing its quality.

Recently, Allflex developed a Tissue Sampling Unit (TSU) for collecting tissue samples without the use of a notcher or biopsy punch. The intended purpose of the TSU system is to collect a clean sample directly into a labeled, sealed tube, which contains a proprietary solution that can hold the tissue for up to a year at room temperature prior to genotyping. If the samples need to be held for longer than a year, they can be frozen directly within the sealed tube.

Because the storage medium in the TSUs is not designed to maintain viable cells, we explored the ability to replace the medium that is part of the TSU system or simply fill TSUs that can be purchased without the proprietary medium. While both methods use the same device (a TSU with Holding Medium), there are differences in processing. When the TSUs intended for genotyping are used, the medium must be removed, the collection tube cleaned and sanitized, and the device resealed. In this instance, the TSU is supplied from Allflex with two sealing balls; one green that is deposited in the collection tube with the sample and one red that seals the end to document that the TSU has been used. When the empty TSUs are purchased (Allflex, No Liquid, Product Number 21315472), they can be sanitized without removal of the genotyping holding medium, but they are not supplied with sealing balls. Instead, a 3 mm ball bearing must be sanitized and inserted into the connector. Regardless of the TSU purchased, a clean ear biopsy can be obtained.

**Figure 6:**
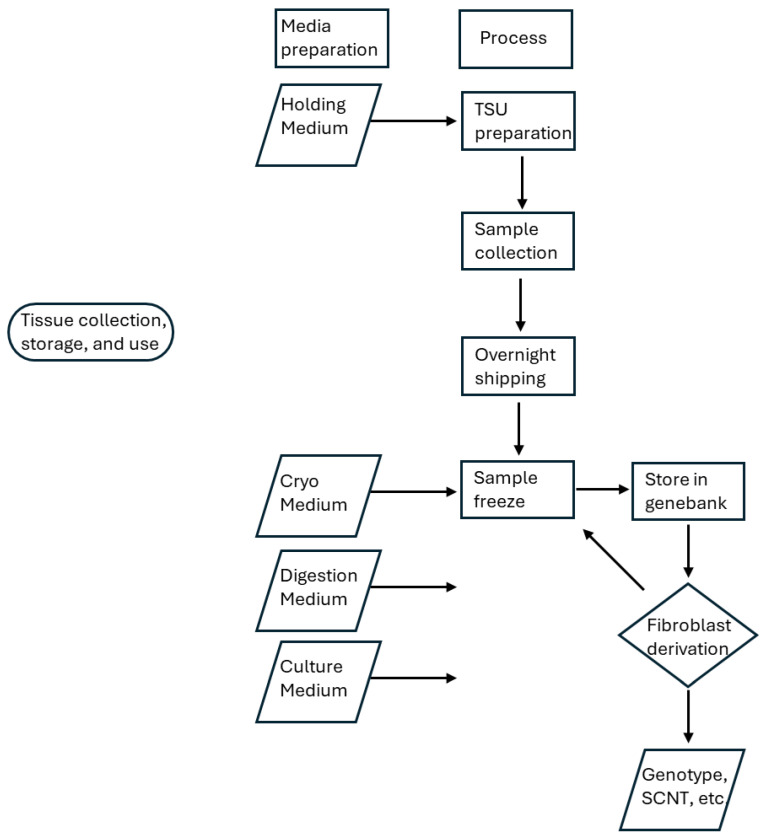
Flowchart for collection, preservation and use of Tissue Sampling Units (TSU).

When the system (Figure 6) was tested on a few sheep ear tissue samples, we demonstrated that successful derivations can be obtained using these methodologies (Table 1). When possible, duplicate samples were collected from an animal on the same day and frozen after holding at 5 °C for up to 3 d. Otherwise, when single samples were collected, they were frozen immediately upon receipt, which is 24 h after collection and storage at 5 °C. The model is certainly not balanced (Table 1) but demonstrates that the protocol is viable for genebanking purposes. In some cases, the quantity of fibroblasts derived is quite substantial and would enable the sample to be split so that a subculture could be used for SCNT or genotyping and the remainder frozen for future uses.

## 5. Conclusions and Limitations

The intent of this Technical Note is to demonstrate an efficient means of collecting, preserving, and utilizing dermal tissue samples for genebanking, genotyping, and SCNT applications. The culture and cryopreservation procedures are well established and produce viable samples, but prior to this research, there was no reported use of the TSU for this purpose. While greater derivation rates were anticipated, samples from 7 of the 11 donor animals (64%) were of a high enough quality to produce a significant quantity of fibroblasts that could be used right away and/or frozen for future uses. We speculate that the low derivation rate may be attributed to a non-optimized cryopreservation process which can be detrimental to cell, tissue, and organ quality [4,5]. Moreover, when the days in holding prior to cryopreservation were statistically analyzed (Proc GLM) [6], there was no significant effect observed (*p* > 0.05). However, the mean quantity of fibroblasts obtained as a function of the number of days in holding prior to cryopreservation declined as the days increased (Day 1 = 1.11, Day 2 = 0.39, Day 3 = 0.23 × 10^6^), indicating that holding time, but not cryopreservation, may be detrimental to derivation success [4,5,7], but not detectable with this small of a data set. Despite this, the use of the TSU is much simpler than other available methods and because of the prelabeled collection tubes (both numbered and QR codes), tracking the samples and matching them to the donor animal is simple and efficient. Still, Groeneveld et al. [1] observed similar results when testing their method on pigs, sheep and goats, and concluded that the methods are successful enough to secure sufficient genetic resources in a cost-effective manner to enable breed repopulation. This is essential for genebank operations that process large batches of samples.

From a through-put perspective, the simplicity of the technique permits a single technician to receive, freeze, and inventory approximately 20–25 samples per hr. In our experiences this speed of processing samples for cryopreservation is a limiting factor for rapidly increasing the size of a genebank collection. However, with the addition of two more technicians and assigning specific tasks to individuals to develop more of an assembly line processing, we were able to increase the productivity of the process and raise our efficiency by five times. This is critical to maximize productivity and minimize the holding time to ensure the highest quality of samples following cryopreservation and thawing.

## Figures and Tables

**Table 1:** Results from tissue culture in 24-well plates using frozen-thawed sheep ear tissue.

Animal	Days in Holding	Days in Culture	Passages	Cells (×10^6^)
Adult ram 1	1	14	2	1.05
Adult ram 2	1	14	3	2.82
Adult ram 2	2	15	0	0
Adult ram 3	1	14	2	1.31
Adult ram 3	1	15	0	0
Adult ram 4	1	14	3	2.64
Adult ram 4	1	14	0	0
Adult ram 5	1	17	2	1.04
Adult ram 6	3	17	1	0.93
Adult ram 7	2	17	2	1.18
Adult ram 8	3	14	0	0
Adult ram 9	2	15	0	0
Ram lamb 1	1	14	0	0
Ram lamb 1	3	15	0	0
Ram lamb 2	3	15	0	0

## Data Availability

All data are presented in the manuscript.
